# Hemp in Animal Diets—Cannabidiol

**DOI:** 10.3390/ani12192541

**Published:** 2022-09-22

**Authors:** Sepideh Fallahi, Łukasz Bobak, Sebastian Opaliński

**Affiliations:** 1 Department of Environmental Hygiene and Animal Welfare, Wrocław University of Environmental and Life Sciences, 51-630 Wrocław, Poland; 2 Department of Functional Food Products Development, Wrocław University of Environmental and Life Sciences, 51-630 Wrocław, Poland

**Keywords:** *Cannabis sativa*, CBD, feed, livestock, poultry

## Abstract

**Simple Summary:**

Plant feed additives have been used in animal diets for improving animal health and welfare. Thus, hemp (*Cannabis sativa*) and its products received attention and much research has been conducted to evaluate the effects of *Cannabis sativa* compounds in animals. Among various substances of this plant, cannabidiol showed desirable effects such as relieving pain and inflammation reduction in some studies. Considering the importance of animal welfare, especially in poultry production, the use of cannabidiol can be effective here.

**Abstract:**

In recent years, interest in hemp use has grown owing to its chemical and medicinal properties. Several parts of this plant, such as seeds, leaves, flowers, and stems are used in medicine, industry, and environmental preservation. Although there were legal restrictions on hemp exploitation in some countries due to the trace presence of THC as a psychoactive element, many countries have legalized it in recent years. Cannabidiol or CBD is a non-psychoactive phytocannabinoid that can activate the endocannabinoid system and its receptors in the central and peripheral nervous system in bodies of different species. Cannabidiol has anti-inflammatory, antioxidative, analgesic, and anti-depressant effects. This review investigates various aspects of cannabidiol use and its potential in animals and humans.

## 1. Introduction

*Cannabis sativa*, generally known as hemp, is an oleaginous angiosperm annual plant from the Cannabaceae family [1]. Its use dates back to 8000 BC [2]. Cultivation of this plant originated in Central Asia (China) [3] and after that expanded across Asia (India, Iran, and Pakistan), South America, Africa, and throughout Europe during medieval times. Hemp has received attention due to its rapid growth rate and high biomass production [2,4]. Every part of this multifunctional plant, such as its seeds, leaves, flowers, fiber and shives, is exploited in the fabric and textile industry, the paper industry, construction, acoustic and thermal insulation (the walls, floors, and roofs), antibacterial detergents, biodegradable plastic, animal bedding, medicine, nutritional supplements, and environmental conservation [5]. This plant’s nutraceutical or health-promoting properties are due to the presence of almost 500 chemical compounds, e.g., flavonoids (cannaflavin and kaempferol), terpenes (limonene and α-pinene), phytocannabinoids (tetrahydrocannabinolic acid, cannabidiolic acid, cannabichromenic acid, and cannabigerolic acid), amino acids, polyunsaturated fatty acids (PUFAs) in the oil pressed from the seeds, minerals, and phenols [6]. Phenolic compounds prevent gastrointestinal disorders [5].

Cannabidiol (CBD) is a phytocannabinoid derived from flowers, leaves and, to a lesser extent, stems. Cannabidiolic acid (CBDA) can change to CBD due to heat exposure [3]. CBD oil was proven to be efficient for therapeutic purposes such as controlling epilepsy, pain and inflammation, anorexia, nausea, anxiety disorders, and insomnia [5,7]. Furthermore, this substance relieves multiple sclerosis pain in humans [8].

Since Yamauchi et al. conducted the first scientific research on the extraction of the chemical components of Cannabis in 1968, interest in the use of industrial hemp and CBD in animal and human nutrition research has grown in recent years [9,10].

The following subjects will be discussed in this review:

- Hemp classification, laws and regulations on its use;
- CBD structure, extraction, and the mechanism of action in the body;
- CBD anti-inflammatory, hepatic and antioxidative effects;
- Hemp in livestock feeding.

## 2. Description of Cannabis

### 2.1. Categorization and Botanical Features

Hemp is included in the Cannabaceae family with four subspecies: sativa, indica, ruderalis, and afghanica [11]. The previous classification categorized Cannabis as just marijuana and hemp, which was a mistake [12]. The difference among the subspecies is because of the interaction of genetics and environment [3], climate, the shape of the plant, and their use [10]. *Sativa* and *indica* are divided into different varieties [1], as demonstrated in Table 1.

Hemp is an angiosperm dioecious plant with a strong root that can enter deeply into the soil [2]. The best growth occurs between 13 and 22 °C in moist nutrient-balanced soils with a pH around six which is rich in nitrogen, potassium, phosphorus, copper, and magnesium, but susceptible to soil compaction [2,3]. Hemp functions well for enhancing soil quality, thus it can be an appropriate choice for use in crop rotation procedures [13]. Staminate plants bloom earlier compared to pistillate ones [14]. Males are taller, whereas females are shorter and have many flowers [10]. Plants cultivated for CBD and oilseed hemp should be kept at some distance to gain better branches and flowers, but fiber hemp has a better stalk growth when planted at high density [13]. Fiber quality is affected by density, infectious fungi, and irrigation [15].

Among several recognized phytocannabinoids, tetrahydrocannabinol (THC) and cannabidiol (CBD) are the two most essential components. THC is a psychoactive compound that is used for recreational purposes. On the other hand, CBD is a non-psychoactive compound known for antioxidant, anti-inflammatory, antipsychotic, anxiolytic, and anticonvulsant effects [16,17]. CBD can prevent oxidative stress and be effective in cancer, diabetes, cardiovascular and neurodegenerative diseases [18].

*Cannabis indica*, mostly known as marijuana or medical Cannabis, is bushier [2] and has intoxicative features which may have medicinal importance [19]. *Cannabis indica* contains a higher level of THC, specifically Δ9-tetrahydrocannabinol (Δ9-THC), whereas *Cannabis sativa*, known as hemp or Industrial Hemp, is taller. The importance of hemp is for the seed and fiber used in several products and their medicinal value [2]. This plant has no intoxicative features. Compared to indica, it has low levels of THC (less than 0.3 wt. %) and higher levels of CBD. BT allele in medical Cannabis encodes tetra-hydrocannabinolic acid synthase, whilst in Industrial Hemp, canabidiolic acid synthase (CBDA) is encoded by BD allele [20]. In a study conducted on female flower transcriptome of hemp and medical Cannabis, an upregulation in the pathway of THC production in medical Cannabis was observed compared to hemp [21]. This can explain the difference between a 10% THC level in medical Cannabis and a 0.3% or less THC level in hemp [22]. In another study, Sawler et al. [23] analyzed 43 hemp and 81 medical Cannabis samples acquired from 14,301 single-nucleotide polymorphisms. This research showed noticeable genetic differences between these two plants—medical Cannabis had a narrow genetics base whilst hemp was more heterogeneous [23].

### 2.2. Laws and Legal Restrictions

In the 1930s, hemp use was prohibited in Canada by the Narcotics Management Act [4,24] due to its previous categorization as being the same type as marijuana. During the Second World War, the United States Department of Agriculture (USDA) persuaded farmers to cultivate hemp [25]. After the war, prohibition was imposed. Simultaneously, hemp was produced to a small extent in some areas of the world, e.g., Eastern Europe, China, Spain, France, and the Soviet Union [26]. The hemp production ban was removed in 1998 in Canada [24].

There is a narrow line for identifying Cannabis as a drug plant or a non-drug one. In the United States and most of European countries including Poland—the maximum THC level for industrial hemp should be 0.3% and 0.2%, respectively [24,27].

As mentioned before, the regulations would be different among the countries related to the therapeutic use of Cannabis (CBD oil extracted from dried flowers) in humans and animals. In Canada, some European countries, and some states of the USA, physicians prescribe many cannabinoid medicines for humans [28]. Based on the European Food Safety Authority (EFSA), hemp seed, hemp expeller, hemp oil, hemp flour and hemp fiber can be utilized in the feed of different animal species. However, this depends on the amount added to the diet [29]. In COMMISSION REGULATION (EU) 2017/1017 of 15 June 2017, the maximum content of THC was set as 0.2% [30].

### 2.3. Nutrients

Various hemp-containing products such as tea, oil, and beer can be used in the food industry [5]. In recent years, hemp seeds, hemp seed cakes, and hemp seed oil have been added to animal feed. Hempseed and hempseed cake can be a good source of protein and fat in the diet, while hemp oil is added to the feed to provide essential fatty acids [5]. Fatty acids and lipids are derived from seeds [10], while cannabinoids, particularly CBD and terpenes, can be extracted from trichomes of flowers in hemp [5].

Seeds are excellent sources of polyunsaturated fatty acids (PUFAs), 20–25% digestible proteins such as albumin and essential amino acids in high levels (arginine, methionine, and cysteine), carbohydrates (25–35%), vitamins, for instance, γ-tocopherol at an amount of 60.85 mg/100 g dry matter, and minerals [4,31,32].

Hempseed oil is rich in PUFAs at an amount of 25–35%, up to 90% consisting of α-linoleic acid (ALA) 18:3 (ω-3) 16%, linoleic acid (LA), 18:2 (ω-6) average 56%, with a 3.5:1 ratio ω-6 to ω-3 [5], γ-linoleic acid (GLA) and stearidonic acid (SDA). Hempseed oil reduces cholesterol levels [33]. Moreover, it contains natural antioxidants [34]. Hemp flowers contain carbohydrates, fiber, vitamins, minerals (Fe, Zn, Cu, and Mn), and essential amino acids [2].

## 3. CBD Structure

### 3.1. Chemical Structure

**Figure 1:**
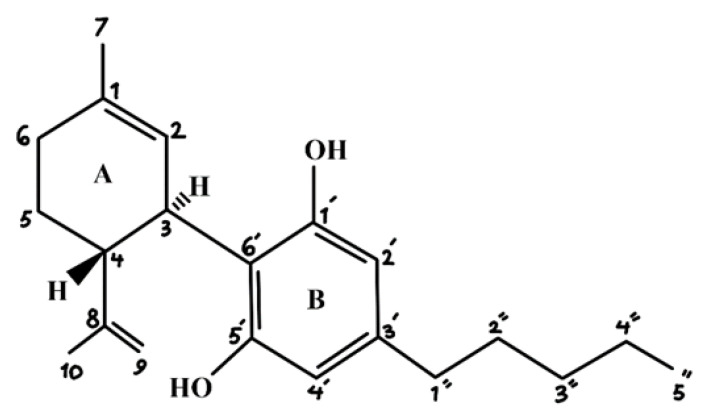
Molecular structure of cannabidiol (CBD).

CBD is a phytocannabinoid with the formula C_21_H_30_O_2_ and a relative molecular mass of 314.464 g. mol^−1^ [35]. The structure of CBD is shown in Figure 1. This molecule consists of a cyclohexene ring (A), a phenolic ring (B), and a pentyl chain. The activity of CBD is attributed to the location of three carbon positions [35]:

(1) In the cyclohexene ring at C-1 with a methyl group,
(2) In the phenolic ring at C-1ʹ and C-5ʹ with a hydroxyl group, and
(3) In the phenolic ring at C-3ʹ with the pentyl chain.

### 3.2. Biosynthesis

**Figure 2:**
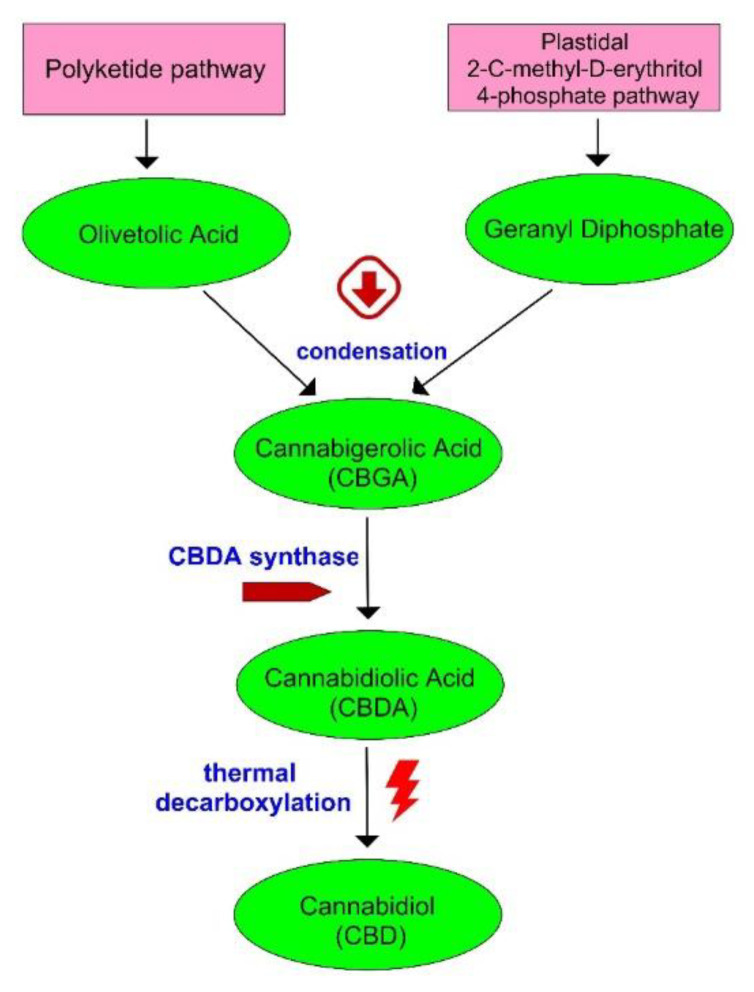
Biosynthesis of CBD.

Cannabidiol is produced in the glandular trichomes of the female hemp flowers [10]. Figure 2 depicts the stages of CBD biosynthesis. Two main precursors (olivetolic acid and geranyl diphosphate) are synthesized in separate pathways [3]. Approximately 95% of the CBD is found in an acidic form such as cannabidiolic acid (CBDA) in fresh biomass [36]. Decarboxylation can be spontaneous, thermal, or alkaline [10]. Terpenes and terpenoids responsible for flavor and aroma can be lost during the decarboxylation process, a disadvantage of decarboxylation [37].

## 4. Methods of Extraction

CBD extraction and purification can be challenging processes due to the presence of THC. The appropriate method should be practical and have the most negligible CBD loss.

**Figure 3:**
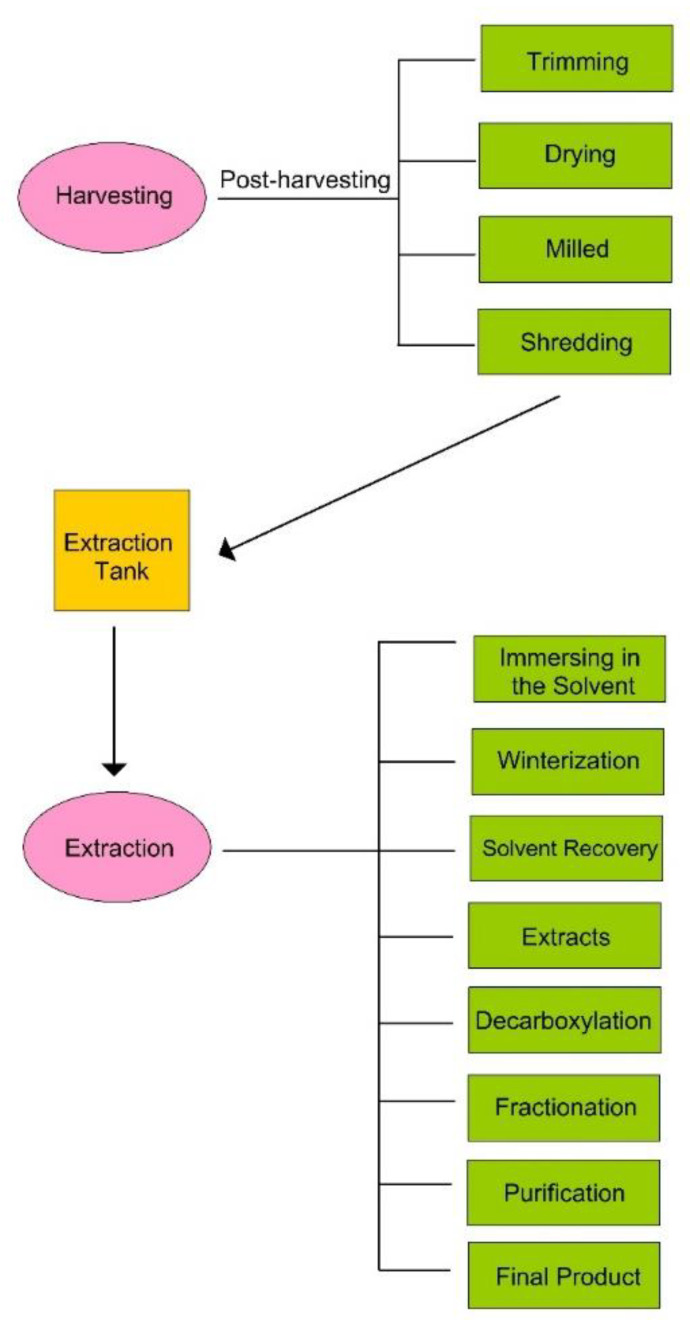
Stages of cannabinoid extraction.

There are different stages in Cannabis processing: selection of variety, cultivation, harvesting, and extraction [10]. In the hemp industry, extraction can be performed from trichomes (chemical extraction) or hempseeds (mechanical extraction) [10]. In the trichome category, cannabinoids and terpenes are extracted. Figure 3 shows the general procedure for cannabidiol extraction. After harvesting, flowers are trimmed (manually or using machines) and should be dried at a low temperature without exposure to the sunlight to inhibit photochemical transformation.

The highest concentration of cannabinoids is found on the flower trichomes surface; hence, mechanical methods such as pressing would not be appropriate for extraction [10]. Intense crushing increases the risk of producing undesirable substances [38]. Organic Solvent Extraction (OSE), Supercritical Fluid Extraction (SFE), and Soxhlet Extraction (SE) can be used mostly as methods of extractions in this category. For extraction processing, polar solvents such as ethanol, methanol, iso-propanol, and dimethyl ether are applied [10]. During the exposure to the solvent, co-extracts such as moisture and heavy residues (heavy metals, pigments, and fatty acids as a black wax) are eliminated. In the winterization stage, the wax is separated below −70 °C, which takes 24 h [39,40]. After winterization, the solvent can be recycled and returned to the process [10].

To obtain a pure product, chromatography, crystallization or distillation can be exploited [40,41]. Mechanical methods such as press extraction and microwave-assisted extraction (MAE) are used for fatty acid extraction from hempseed oil. Various chemical CBD extraction methods are described in the following subsections.

### 4.1. Soxhlet Method

This method has been widespread for plant oil extraction [42] and some organics such as vanillin [43], coffee [44], marijuana cigarette [45], and orange juice [46]. In this method, the substance is constantly exposed to a stream of the solvent during the operation. Moreover, the particle size of the substance should be considered. This method is undesirable currently due to the excessive solvent and energy needs, making it a less popular method today [47,48].

Some researchers used the Soxhlet method to extract some compounds from hemp. Pandohee et al. derived cannabinoids from Cannabis with the Soxhlet method in consecutive batches of ethyl acetate. Before the operation, roots and leaves were grounded and treated under 368 µm. In the first batch, 0.5 g of the powder and 300 mL of ethyl acetate were made at 78 °C for 1.5 h. The second batch consisted of extraction repetition with 300 mL of solvent for 1 h, and finally, the combination of extractions vacuumed at 40 °C [49]. Chang et al. extracted cannabinoids from hemp seed with 300 mL methanol for 8 h at 90 °C and cooled at 15 °C afterwards [50].

### 4.2. Immersion Method (Maceration)

The substance is immersed in the solvent for a period of time. Generally, this term is used for conventional extraction methods, including OSE methods. The extraction method is named according to the used solvent. Ethanol and methanol solvents, for instance, are widespread for cannabinoid extraction due to their polar properties and boiling point. However, ethanol is preferable due to its lower toxicity [10].

Soxhlet and Immersion methods caused some safety and environmental concerns; thus other methods are applied for effective extraction.

### 4.3. Supercritical Fluids Extraction (SFE)

Different fluids are applied in the SFE process, such as methanol, ethanol, carbon dioxide, water, sulfur hexafluoride, nitrous oxide, and n-pentane [51]. Since CO_2_ is non-toxic, non-flammable, and affordable, it has been employed extensively in the SFE method [52,53]. To obtain an efficient extract, pressure and temperature increase to the critical condition of CO_2_ (P_c_ = 7.38 MPa, T_c_ = 30.98 °C). Recovering and reusing carbon dioxide during the process is another advantage of the SC-CO_2_ method. Furthermore, co-solvents moderate the solute and solvent and produce more efficient extraction [10]. Water, acids [54], esters, ketones, alcohols, and aldehydes [55] are employed as co-solvents.

The supercritical extraction method is expensive, so the OSE method is still more commonly in use. SC-CO_2_ has a medium to low potential for extraction of cannabinoids and terpenes [10]. However, ethanol has a greater solubility range—cannabinoid and terpene solubility decreased on winterization and a reduction in temperature and, consequently, there were more sediments [10].

Grinding, ultrasonication, and high-pressure homogenization are applied as pretreatment methods (the two latter ones are used at a small scale) for both cannabinoids and lipids [38,50,56].

### 4.4. Supercritical Hot Water Extraction

In this method, pressurized hot water is employed as a solvent with equivalent solvability features to ethanol and methanol [57]. It has been shown that this method can be faster for extracting CBD, cannabichromene (CBC), cannabigerol (CBG), cannabinol (CBN), and THC from hempseed compared with the older and traditional methods [58]. In contrast with the SC-CO_2_ method, supercritical hot water can be more expensive, impose more energy on the system, and has safety problems due to the water supercritical condition (T_c_ = 373.94 °C, P_c_ = 22.064 MPa) [10].

### 4.5. High-Performance Liquid Chromatography (HPLC)

This technique can be used for the separation and identification of CBD. This technique consists of a column with a diameter of 2 ÷ 4.6 mm and a length of 20 ÷ 250 mm, with different modifications in the surface stationary phase [59]. The detector can be UV (ultraviolet) or DAD (diode-array detection). Among LC columns, reverse-phase C18 (less often C8) packed columns are the most common for analysis of cannabinoids. CBD was detected in *C. sativa* with reverse-phase HPLC [60]. Moreover, Mandrioli et al. reported cannabinoid detection using HPLC-UV with a conventional C18 column [61]. In another study, HPLC-DAD was used to determine CBD content [62].

## 5. Mechanism of Action

### Endocannabinoid System (ECS)

The endocannabinoid system is a type of endogenous signaling system adjusted by sleep, stress levels, physical activity, and food which maintains homeostasis in the body. Endocannabinoids include amides, ethers, and esters from long-chain polyunsaturated fatty acids (PUFAs) [35]. ECS can be found in invertebrates [63] and vertebrates, e.g., amphibians (frog), zebrafish [64], poultry (chickens) [64], and mammals [65]. In dogs, cannabinoid receptors or their ligands can be found in the central and peripheral nervous system, embryo, skin, the gastrointestinal tract [66,67,68,69] and also in the brain, skin, ovary, and oviduct in cats [70,71,72]. ECS presents in some body tissues and is efficient in alleviating pain, memory, appetite, anti-inflammatory responses, immunosuppression, sleep regulation, reproductive functions [5], and a reduction in oxidative stress [35]. The main parts of the endocannabinoid system [73,74] are depicted in Table 2.

Due to the resemblance between prostaglandins and endocannabinoid structures, interaction between metabolic pathways occurs [81]. THC is an agonist for CB1 receptors but, in some cases, it acts as an antagonist and interacts with CB2 receptors [79,80]. Interaction between THC and CB1 receptors can prevent the release of neurotransmitters. CBD is a non-psychoactive compound, so it has a low direct effect on CB1 and CB2 receptors.

There are 76 types of molecular CBD targets [35]. CBD can directly or indirectly interact with different receptors, enzymes, and ion channels in the endocannabinoid system [82]. CBD enhances endocannabinoid expression. Therefore, it indirectly affects inflammation and redox balance [83]. Among the critical CBD targets is nuclear receptors, e.g., peroxisome proliferator-activated receptor gamma (PPAR-γ), which is involved in the expression of genes that control inflammation [81]. Direct CBD activity is increased by the action of AEA and 2-AG [84]. Among enzymes, CBD interacts with various cytochrome P450 (CYP) enzymes involved in drug metabolism [81]. CBD can stimulate calcium ions and adjust calcium ion homeostasis in immune and inflammatory cells, which is essential for pro-inflammatory cytokine secretion [85]. These cytokines, such as interleukin-1 (IL-1), interleukin-6 (IL-6), and TNF-α, are necessary for balancing the immune system.

## 6. Analgesic and Anti-Inflammatory Effects of CBD

Numerous dose-dependent studies on the pain-controlling effects of hemp oil and CBD were conducted on some animal species and humans. Since pain and inflammation exist simultaneously in many conditions, various studies show that CBD has analgesic potential, and anti-nociceptive and anti-inflammatory effects on some painful diseases and disorders.

In veterinary medicine, CBD has been used to alleviate cancer pain, osteoarthritis, neuropathic pain, and mood disorders in dogs and cats [86,87]. Moreover, it has been proved that CBD has mild side effects (decreasing appetite, nausea, and sedation) in human clinical studies [88,89,90].

The results of research on CBD’s anti-nociceptive and anti-inflammatory effects in recent years are shown in Table 3. Furthermore, some definitions are described in the following for a better understanding.

Based on the research results depicted in Table 3, it is noticeable that CBD is efficient in reducing pain and inflammation. There is no specific study on the effectiveness of CBD in controlling pain in livestock production, including poultry. Broilers are susceptible to disorders and abnormalities due to their fast growth rate [105]. In most cases, these disorders are accompanied by pain and inflammation. Additionally, layers are prone to bone fracture during the laying period, particularly keel fracture [106]. These problems are significant concerns in terms of poultry welfare at the industrial scale. As it is proved that CBD has anti-nociceptive effects, future studies on its efficiency are required in the poultry industry.

## 7. Hepatotoxicity and Tolerability

In recent years, studies have been conducted to investigate the effects of different parts of the hemp plant (e.g., seeds) and CBD on liver function in humans and animals. The results of research investigations related to the effect of Cannabis on the liver are depicted in Table 4.

Since hemp is a source of unsaturated lipids, it is prone to oxidation [111]. The liver metabolizes lipids and absorbs portomicron in chickens [112,113]. The peroxidation of lipids causes oxidative damage and hepatic diseases in laying hens. Moreover, any injuries to hepatic cells have considerable repercussions on the mineral metabolism (Ca and P, for instance), affecting eggshell quality and skeletal structure [114]. Thus, measuring biochemical parameters in plasma or serum can indicate whether the liver functions properly [115,116]. Table 5 shows the effect of CBD on liver performance in different species.

Samara et al. showed that CBD can inactivate cytochromes P4503A (CYP3A) and P4502C in hepatic drug metabolism [120]. Cytochrome P4503A is involved in drug metabolism in the liver and gastrointestinal tract, and cytochrome P4502C is responsible for xenobiotic oxidation. Long-term CBD use has been shown to stimulate the CYP3A and CYP2B10 enzymes in mice livers [121]. CBD inhibits the absorption of carcinogenic substances in blood and protects DNA [122]. On the other hand, Ewing et al. analyzed the gene expression for hepatotoxicity [119]. They reported that CBD regulates more than 50 genes. These genes are responsible for oxidative stress, drug-metabolizing enzymes, and pathways related to lipid metabolism. Based on the results of this study, CBD showed signs of hepatotoxicity. An increase in drowsiness and a reduction in anxiety on use of higher doses of CBD (300 mg oral) were observed in humans. On the other hand, intoxication was not reported at lower doses (30 mg oral) [80].

Based on the results presented in the previous paragraph, it seems that higher levels of CBD result in more side effects and the risk of liver damage. Since CBD is a dose-dependent substance, it has shown different physiological responses. Thus, it is essential to indicate the optimal dose in different species.

## 8. CBD Health Benefits

Several studies showed that CBD has beneficial effects on nervous system diseases and mental health. Hypoxic-ischemic (HI) is a brain injury that occurs due to oxygen deprivation in brain cells and causes neurological impairment, e.g., decreased cognitive function and epilepsy [3]. In a study on mice conducted by Castillo et al. [123], it has been demonstrated that CBD increases neuroprotection in mouse brain ischemia conditions. Further, it boosts the reconstruction of the hippocampus [124]. The endocannabinoid system has a function in emotional response and behavior [125]. Shbiro et al. [126] demonstrated that CBD can be used as an anti-depressant in depressive mice.

In the nervous system, the brain consumes a high amount of oxygen [127]. Oxygen is among the most crucial elements in organisms. However, oxygen can be very harmful when it generates oxygen free radicals or generally ROS (reactive oxygen species), which result in damage to DNA, RNA, and proteins as well as cell death. The lack of a balance between oxidants and antioxidants causes oxidative stress. Oxygen reduction (O_2_) produces a superoxide that is a precursor to many reactive oxygen species such as hydrogen peroxide (H_2_O_2_). Due to the high lipid content in the brain, it is sensitive to oxidation [127]. It is proven that CBD has antioxidant activity, which can be direct or indirect [35].

### 8.1. Direct Antioxidant Activity

CBD affects the elements in the redox system. CBD adjusts the activity and level of oxidants and antioxidants [128,129]. In addition to catching free radicals and inhibiting their chain reaction, CBD can prevent producing superoxide radicals and decrease ROS activity. The antioxidant activity of CBD results from the activation of nuclear erythroid 2-related factor or Nrf2 (a redox-sensitive transcription factor) [130]. This factor can transcript cytoprotective genes such as antioxidant genes [131]. The antioxidant activity of CBD is generally due to the hydroxyl group of the phenol ring [132]. Wu et al. reported that CBD use can increase the amount of GSH (glutathione) in microglia cells of mice [133]. GSH acts with vitamins A, C, and E [134]. CBD has 30–50% more antioxidant activity in comparison with α-tocopherol or vitamin C [16].

Lipid peroxidation is among the most prominent processes that occur in the body which causes polyunsaturated fatty acid (PUFA) oxidation [135]. When ROS reacts with PUFAs, lipid hydroperoxides are made. Oxidative fragmentation produces unsaturated aldehyde such as malondialdehyde (MDA) [136]. Sun et al. showed that CBD can decrease lipid peroxidation in hippocampal neuronal cells of mice (HT22) when they experience oxygen and glucose depletion under reperfusion conditions [137].

### 8.2. Indirect Antioxidant Activity

Some molecular compounds have a role in the redox system. CBD can indirectly interact with these molecules and play a role in regulating redox balance. Anandamide (AEA) can be increased due to the effect of CBD on the activity of the endocannabinoid system [17]. Since AEA is a fatty acid neurotransmitter, an increase in this substance affects the interaction between cannabinoids and receptors [138]. Activation or prevention of the activity of CB1 and CB2 receptors can depend on CBD concentration [139].

## 9. Hemp Use in Livestock Diet

Much research has been performed on adding hemp to animal feed. In recent years, attention has been paid to *Cannabis sativa* use in farm animal diets, especially poultry, due to its positive effects. It is important to investigate the effects of the different components of this plant on livestock and poultry in future research.

The results of studies on hemp use in laying hens show (Table 6) that hemp oil and hempseed are mainly responsible for increasing n-3 PUFA levels in egg yolk.

Table 7 demonstrates hemp use effects on broilers. Bone fractures cause mortality in intensive poultry farming. It has been shown that *Cannabis sativa* and its metabolites increase tibia strength and decrease the deformation rate in broilers and laying hens.

There are studies regarding Cannabis inclusion in other animal species, as shown in Table 8. Hemp products increased linoleic and linolenic acids in quail meat and eggs. Moreover, a higher amount of conjugated fatty acid and PUFAs was observed in goat milk.

Based on the research results of Kleinhenz et al. [149], the biomarkers of inflammation and stress declined on adding hemp to male Holstein diets (with a target dose of 5.5 mg/kg CBDA).

There is a gap in the literature on CBD use, specifically in poultry. Since CBD is an efficient substance in the Cannabis plant, its effects in further studies should be taken into account.

## 10. Conclusions

Cannabis has been utilized widely in recent years. Hemp oil, hempseed oil, and hempseed cake improved performance and bone strength, enriched egg fatty acid profiles, and increased milk yield in livestock production. CBD use as a non-psychoactive compound showed promising results in alleviating and preventing pain, oxidation, inflammation, and anxiety in different species of animals and also in humans. An appropriate method of extraction for a high level of purity and correct dosage of this substance is important in terms of hepatic conditions. Even though CBD has been used in several animal studies, the absence of research on CBD use in poultry is noticeable. Since welfare is a major concern in the poultry industry, evaluating the effects of CBD in further research should be considered.

## Figures and Tables

**Table 1:** Different varieties of Cannabis [1].

Species	Varieties	
	Domesticated	Wild
*Cannabis sativa*	*sativa*	*spontanea*
*Cannabis indica*	*indica*	*kafiristanica*

**Table 2:** Different parts of the endocannabinoid system.

Main units	Description	Functions	Compounds
Endocannabinoids	Lipid compounds metabolized in multiple enzymatic pathways	Binds to the receptors, recovers homeostasis after cellular stress, interacts with the human endocannabinoid system [75,76]	Neurotransmitters: anandamide-N-arachidonylethanolamine (AEA) and 2-arachidonylglycerol (2-AG)
Cannabinoid receptors		Able to stimulate receptors, produces physiological responses	G protein-coupled receptors (GPCRs), also known as 7-transmembrane receptors (7-TM receptors) and their endogenous ligands [3]
CB1 receptors	Found in the central nervous system, e.g., brain and spinal cord, some cells in the immune system, muscles, liver, kidney, lungs, reproductive system, and adipose tissues	Mediates the release of neurotransmitters: acetylcholine, noradrenaline, dopamine, gamma-aminobutyric acid (GABA), and glutamate Increases reactive oxygen species (ROS) production and pro-inflammatory responses such as tumor necrosis factor-alpha synthesis (TNF-α) [77]	
CB2 receptors	Active receptors in the peripheral nervous system, e.g., immune system, liver, kidney, adipose tissues, and spleen	Releases cytokines, adjusts immune cell migration [78,79,80] without psychoactive functions [53], decreases ROS, (TNF-α) levels, oxidative stress, and inflammation [77]	
Enzymes		Involved in synthesis and catabolism [81]	Fatty acid amide hydrolase (FAAH), monoacylglycerol lipase

**Table 3:** Anti-nociceptive and anti-inflammatory effects of CBD in different species.

Species	Type of Pain or Disorder	CBD Amount and Duration	Results	Reference
Dogs (16)	OA ^a^ pain and lameness	CBD oil ^b^ 2 mg/kg every 12 h, 4 weeks	Significant reduction in pain, no significant difference in lameness degree	[91]
Dogs (37)	Chronic maladaptive pain	CBD oil ^c^ 0.25 mg/kg once a day for 3 days, then every 12 hrs	Significant reduction in pain, increase in mobility and quality of life	[92]
Dogs (20)	OA pain	20 mg/day (0.5 mg/kg) naked CBD ^d^, 50 mg/day (1.2 mg/kg) naked CBD, 20 mg/day liposomal CBD ^e^	Significant reduction in pain, increase in mobility	[93]
Rats	RA ^f^ pain	CBD gels ^g^ (0.6, 3.1, 6.2 or 62.3 mg/day) for 4 days after arthritis	Significant decrease in joint swelling, inflammation biomarkers, pain scores, and synovial membrane thickness	[94]
Rats	Spared nerve injury	Repeated CBD injections ^h^ (0.1–1.0 mg/kg)	Declined mechanical allodynia, anxiety-like behavior	[95]
Rats	Exposed to pain (paw pressure and tail-flick test)	Intra-ventrolateral periaqueductul grey (PAG) microinjections of CBD (1.5, 3 and 6 nmol)	Reduction in activity of ON and OFF neurons, anti-nociceptive responses in the tail-flick test	[96]
Mice	Sciatic nerve injury (neuropathic pain ^i^)	CBD-containing gelatine ^j^, orally ad libitum	Significant reduction in pain after 3 weeks of surgery	[97]
Mice	Type 1 diabetes	5 mg/kg CBD ^k^, 5 times weekly for 10 weeks	Significant reduction in leukocyte activation, increase in pancreatic microcirculation	[98]
Mice	Spinal cord injury	Intraperitoneal injections of CBD ^l^ 1.5 mg/kg, for 10 weeks following injury	Reduction in pro-inflammatory cytokine, prevented thermal sensitivity development	[99]
Human	Peripheral neuropathic pain	Transdermal CBD (250 mg CBD/3 fl. Oz)	Significant reduction in severe pain, cold and itchy sensations	[100]
Human (72 children, 60 adults)	Treatment-resistant epilepsy	5 mg/kg/day CBD ^m^, titrated up to 50 mg/kg/day	Reduction in seizure frequency and severity	[101]
Human	Dravet syndrome ^n^	100 mg/mL CBD ^o^, oral solution at 2–10 mg/kg/day, titrated up to 25–50 mg/kg/day. Evaluating seizures at 12-week intervals through 96 weeks CBD ^p^ 20 mg/kg of body weight per day + standard antiepileptic treatment	Decrease in motor seizures and improved patients conditions	[102,103]

^a^ Osteoarthritis causes joint degeneration, which is found both in humans and animals. Non-steroidal anti-inflammatory drugs (NSAIDs) and opioids are used for alleviating pain [81]. ^b^ Final desiccated CBD reconstituted into an olive oil base. ^c^ Delivered on food. ^d^ Solubilized in coconut oil. ^e^ With a sunflower lecithin base, each liposome encapsulated 10 to 20 mg/mL CBD. ^f^ Rheumatoid arthritis is an autoimmune and inflammatory disease that affects synovial tissue and generates joint inflammation and hyperplasia [104]. ^g^ Dissolved in ethanol, gel containing 1 or 10% CBD rubbed into the skin. ^h^ Prepared in a vehicle of ethanol/Tween 80/0.9% saline (3:1:16), injected intravenously (I.V.). ^i^ Neuropathic pain is a chronic pain caused by damage to a nerve or some diseases (e.g., type 1 diabetes and MS) that affects the somatosensory nervous system and is difficult to alleviate [81]. ^j^ Dissolved into 95% EtOH to a concentration of 20 mg/mL, then added to obtain a final concentration of 1 mg/15 mL (g) of gelatin. ^k^ CBD ≥99% purity, intraperitoneal injection (I.P.). ^l^ Dissolved with a 1:1:18 ratio of anhydrous ethanol, cremophor, and 0.9% saline. ^m^ Highly purified CBD in sesame oil (100 mg/mL; Epidiolex^®^) orally. ^n^ Dravet syndrome is drug-resistant epilepsy that starts during the first year of life [81]. ^o^ Highly purified CBD (100 mg/mL), oral solution (Epidiolex^®^). ^p^ 100 mg CBD/mL.

**Table 4:** The effects of using hemp on liver function in poultry.

Species	Experiment Duration	Hemp Type and Amount	Results	Reference
19-week-old Bovan White	12 weeks	HS ^a^: 10, 20% HSO ^b^: 4, 8, 12%	Significant decrease in the expression of hepatic fatty acid desaturase 1 and 2 (genes for the desaturation of polyunsaturated fatty acids)	[107]
19-week-old Lohmann LSL- Classic White	12 weeks	HS: 10, 20, 30% HSO: 4.5, 9.0%	No significant effects on proteins, glucose, uric acid, and cholesterol plasma levels HS 10, 20% and HSO 4.5%: The significant lowest level of the gamma-glutamyl transferase, reduction in liver damage HS: Significant decrease in AST ^c^ levels, possible protective effect of HS (10, 20%) and HSO (4.5%) on liver damage	[108]
1-day-old male Ross 308	35 days	EF ^d^: 6% HS: 3, 4, 5% HS + EF: (3% and 6%, 4% and 6%, 5% and 6%)	HS + EF (5 and 6%): Positive increase in the vitamin E level in the liver	[109]
1-day-old Caribro-Vishal	42 days	HS: 0.2% HS + DS: ^e^ (0.2% and 0.3) HS: 0.3% HS + DS: (0.3% and 0.3) BMD ^f^: 0.025%	Significant reduction in triglyceride, LDL ^g^ and total cholesterol levels, significant decrease in AST and ALT ^h^, improvement in serum lipid and liver enzyme levels	[110]

^a^ Hempseed, ^b^ hempseed oil, ^c^ aspartate transaminase, ^d^ extruded flaxseed, ^e^ dill seed, ^f^ bacitracin methylene disalicylate, ^g^ low-density lipoprotein, and ^h^ alanine transaminase.

**Table 5:** CBD use effects on liver function in pets and mice.

Species	Health Status	CBD Amount and Duration	Results	Reference
Dogs	OA pain and lameness	CBD oil: 2, 8 mg/kg every 12 h 4 weeks	A significant increase in serum ALP ^a^ No observable side effects	[91]
Dogs	Idiopathic epilepsy	CBD-infused oil: 2.5 mg/kg twice daily 12 weeks	A significant increase in serum ALP	[117]
Dogs and cats	Healthy	CBD chews: 2 mg/kg orally twice daily 12 weeks	No significant changes in serum chemistry Safe in dogs Adverse effects of excessive licking and head shaking for cats	[118]
8-week-old mice	Healthy	CBD extract: (acute toxicity, 24 h) 246, 738, or 2460 mg/kg (sub-acute toxicity, daily doses) 61.5, 184.5, or 615 mg/kg for 10 days	2460 mg/kg: A significant increase in LBW ^b^, plasma ALT ^c^, AST ^d^, and total bilirubin, evidence of hepatotoxicity 615 mg/kg: A moderate increase in LBW, ALT, AST, and total bilirubin	[119]

^a^ Alkaline phosphatase, ^b^ liver-to-body weight, ^c^ alanine aminotransferase, and ^d^ aspartate transaminase.

**Table 6:** Study results on hemp use in laying hens.

Laying Hens	Experiment Duration	Hemp Type and Amount	Results	Reference
19-week-old Lohmann white	6 weeks	HO ^a^ or HΩ ^b^: 4 or 8%	Significant increase in total n-3 PUFAs ^c^ and a significant reduction in MUFAs ^d^ (both in egg yolks) in all groups No effect on performance and egg yolk n-6 PUFAs	[140]
19-week-old Bovan white	12 weeks	HO: 4, 8, 12% HS ^e^: 10, 20%	20% HS: Significantly increased egg weight Significant increase in the total egg yolk n-3 fatty acid content No effect on average hen-day egg production	[107]
30-week-old Bovan white	19 weeks	HSC ^f^: 10, 20, 30%	Significant reduction in body weight in all treatments No effect on performance	[141]
30-week-old Bovan white	3-week acclimation phase + 16 weeks	HSC: 10, 20, 30%	Significant increase in egg PUFAs No detectable cannabinoid residue level in eggs, blood, breast meat, body fat, liver, kidneys and spleen	[142]
Lohmann Brown	12 weeks	HS: 3, 6, 9%	3% HS: Significantly increased egg production and mass 9% HS: Significantly decreased egg shell thickness Positive effect on tibia Ca ^g^ concentration Significant decrease in egg yolk cholesterol Significant increase in breaking strength of tibia in all groups	[143]

^a^ Hemp oil, ^b^ hemp omega, ^c^ polyunsaturated fatty acids, ^d^ monounsaturated fatty acids, ^e^ hempseed, ^f^ hempseed cake, and ^g^ calcium.

**Table 7:** The impact of Cannabis use on broilers.

Broilers	Experiment Duration	Hemp Type and Amount	Results	Reference
1-day-old male Ross 308	From days 9 to 35 (challenge with *Clostridium perfringens*)	HE ^a^: 15 g/kg (12% CBD)	Upregulation in gene expression involved in gut barrier function Increase in the activity of gut bacterial enzyme	[144]
150-day-old mixed-sex Ross 308	21 days	HO or HΩ: 3 or 6%	Significant increase in total n-3 PUFAs in thighs and breasts Significant reduction in MUFAs in thighs No effect on performance and meat n-6 PUFAs	[140]
1-day-old male Ross 308	35 days	EF ^b^: 6% HS: 3, 4, 5% HS + EF: (3% and 6%, 4% and 6%, 5% and 6%)	HS + EF (4% and 6%): Significantly increased body weight, decreased n-6/n-3 fatty acid ratio in breast meat HS, HS + EF (40 and 60 g/kg), HS + EF (50 and 60 g/kg): Positive effect on bone strength	[109]
1-day-old male Ross 308	6 weeks	HS: 2.5, 5, 7.5% DOS ^c^: 0.1%	HS 2.5%: Significant reduction in average daily feed intake and ADG ^d^ HS and DOS: No significant effect on complete blood count, antibody production and relative weight of bursa and spleen	[145]
1-day-old Caribro-Vishal	42 days	HS: 0.2%, HS + DS ^e^: (0.2% and 0.3) HS: 0.3% HS + DS: (0.3% and 0.3) BMD ^f^:0.025%	Significant reduction in Coliform count in caecum and jejunum No effect on performance, jejunal villus height and crypt depth	[110]

^a^ Hemp extract, ^b^ extruded flaxseed, ^c^ dextran oligosaccharide, ^d^ average daily gain, ^e^ dill seed, and ^f^ bacitracin methylene disalicylate.

**Table 8:** The effect of hemp use on different animal species.

Other Species	Experiment Duration	Hemp Type and Amount	Results	Reference
7-day-old Japanese quails	5 weeks	HS: 5%, 10%, 20%	20% HS: Significant decrease in breast meat cooking loss Significant reduction in palmitoleic and oleic FAs in breast meat Significant increase in meat linoleic and linolenic acid	[146]
8-week-old laying quails	6 weeks	HS: 5%, 10%, 20%	Significant linear increase in egg linoleic and linolenic FAs Significant decrease in egg palmitoleic and oleic FAs	[146]
Swedish red dairy cows	5 weeks, 1 week (pre-experimental period)	HSC: 14.3, 23.3, 31.8% (dry matter)	14.3% HSC: Higher milk yield 23.3 or 31.8% HSC: No benefits in milk performance	[147]
Steers	166 days	Full-fat HS: 9 or 14%	Significant increase in CLA ^a^ level, also trans and saturated fats in tissues No effect on DMI ^b^, ADG, carcass traits	[148]
Male Holstein cattle	14 days	IH ^c^: 25 g mixed in 200 g of grain (target daily dose of 5.5 mg/kg CBDA ^d^)	Significant increase in lying behavior Significant decrease in cortisol level and PGE_2_^e^	[149]
Male Holstein calves	Single oral dose, 4 days	IH: 35 g (target dose of 5.4 mg/kg CBDA)	No significant changes in serum parameters	[150]
Carpathian goats	31 days	HSO ^f^: 93 g/day	Higher milk fat content, increase in conjugated fatty acid and PUFAs No effect on milk yield	[151]
Pregnant sows	10 days (before farrowing), 21 days (lactation period)	HS: 2% (10 days) 5% (21 days)	Significant improvement in sows oxidative status during lactation Positive effect on antioxidant enzyme activities (TAC ^g^, NO ^h^) Significant decrease in plasma lipid peroxidation until weaning	[152]

^a^ Conjugated linoleic acid, ^b^ dry matter intake, ^c^ industrial hemp (*Cannabis sativa*), ^d^ cannabidiolic acid, ^e^ prostaglandin E_2_, ^f^ hempseed oil, ^g^ total antioxidant capacity, and ^h^ nitric oxide production.

## Data Availability

Not applicable.

## References

[1] Small E., Cronquist A. (1976). A practical and natural taxonomy for Cannabis. Taxon.

[2] Rehman M., Fahad S., Du G., Cheng X., Yang Y., Tang K., Liu L., Liu F., Deng G. (2021). Evaluation of hemp (*Cannabis sativa* L.) as an industrial crop: A review. Environ. Sci. Pollut. Res. Int..

[3] Vasantha Rupasinghe H.P., Davis A., Kumar S.K., Murray B., Zheljazkov V.D. (2020). Industrial Hemp (Cannabis sativa subsp. sativa) as an Emerging Source for Value-Added Functional Food Ingredients and Nutraceuticals. Molecules.

[4] Callaway J.C. (2004). Hempseed as a nutritional resource: An overview. Euphytica.

[5] Della Rocca G., Di Salvo A. (2020). Hemp in Veterinary Medicine: From Feed to Drug. Front. Vet. Sci..

[6] Russo E.B. (2011). Taming THC: Potential cannabis synergy and phytocannabinoid-terpenoid entourage effects. Br. J. Pharmacol..

[7] Shannon S., Opila-Lehman J. (2016). Effectiveness of Cannabidiol Oil for Pediatric Anxiety and Insomnia as Part of Posttraumatic Stress Disorder: A Case Report. Perm. J..

[8] Rajesh M., Mukhopadhyay P., Bátkai S., Patel V., Saito K., Matsumoto S., Kashiwaya Y., Horváth B., Mukhopadhyay B., Becker L. (2010). Cannabidiol attenuates cardiac dysfunction, oxidative stress, fibrosis, and inflammatory and cell death signaling pathways in diabetic cardiomyopathy. J. Am. Coll. Cardiol..

[9] Yamauchi T., Shoyama Y., Matsuo Y., Nishioka I. (1968). Cannabigerol Monomethyl Ether, a New Component of Hemp. Chem. Pharm. Bull..

[10] Valizadehderakhshan M., Shahbazi A., Kazem-Rostami M., Todd M.S., Bhowmik A., Wang L. (2021). Extraction of Cannabinoids from *Cannabis sativa* L. (Hemp)—Review. Agriculture.

[11] McPartland J.M., Clarke R.C., Watson D.P. (2000). Hemp Diseases and Pests: Management and Biological Control—An Advanced Treatise.

[12] Aiello G., Fasoli E., Boschin G., Lammi C., Zanoni C., Citterio A., Arnoldi A. (2016). Proteomic characterization of hempseed (*Cannabis sativa* L.). J. Proteom..

[13] Adesina I., Bhowmik A., Sharma H., Shahbazi A. (2020). A review on the current state of knowledge of growing conditions, agro-nomic soil health practices and utilities of hemp in the United States. Agriculture.

[14] Amaducci S., Scordia D., Liu F.H., Zhang Q., Guo H., Testa G., Cosentino S.L. (2015). Key cultivation techniques for hemp in Europe and China. Ind. Crops Prod..

[15] García-Tejero I.F., Durán-Zuazo V.H., Pérez-Álvarez R., Hernández A., Casano S., Morón M., Muriel-Fernández J.L. (2014). Impact of plant density and irrigation on yield of hemp (*Cannabis sativa* L.) in a Mediterranean Semi-arid Environment. J. Agric. Sci. Technol..

[16] Iffland K., Grotenhermen F. (2017). An Update on Safety and Side Effects of Cannabidiol: A Review of Clinical Data and Relevant Animal Studies. Cannabis Cannabinoid Res..

[17] Lim K., See Y.M., Lee J. (2017). A Systematic Review of the Effectiveness of Medical Cannabis for Psychiatric, Movement and Neu-rodegenerative Disorders. Clin. Psychopharmacol. Neurosci..

[18] Smeriglio A., Giofrè S., Galati E.M., Monforte M.T., Cicero N., D’Angelo V., Grassi G., Circosta C. (2018). Inhibition of aldose re-ductase activity by Cannabis sativa chemotypes extracts with high content of cannabidiol or cannabigerol. Fitoterapia.

[19] Grotenhermen F., Russo E., Zuardi A.W. (2017). Even high doses of oral cannabidol do not cause THC-like effects in humans: Comment on Merrick et al. Cannabis and Cannabinoid Research 1:102–112. https://doi.org/10.1089/can.2015.0004. Cannabis. Cannabinoid. Res..

[20] De Meijer E.P.M., Bagatta M., Carboni A., Crucitti P., Moliterni V.M.C., Ranalli P., Mandolino G. (2003). The inheritance of chem-ical phenotype in *Cannabis sativa* L.. Genetics.

[21] Van Bakel H., Stout J.M., Cote A.G., Tallon C.M., Sharpe A.G., Hughes T.R., Page J.E. (2011). The draft genome and transcriptome of Cannabis sativa. Genome Biol..

[22] Russo E.B. (2007). History of cannabis and its preparations in saga, science, and sobriquet. Chem. Biodivers.

[23] Sawler J., Stout J., Gardner K.M., Hudson D., Vidmar J., Butler L., Page J.E., Myles S. (2015). The genetic structure of marijuana and hemp. PLoS ONE.

[24] Agriculture and Agri-Food Canada (2020). Crop profile for Industrial Hemp in Canada. https://www.publications.gc.ca/site/eng/9.903431/publication.html.

[25] Shahzad A. (2012). Hemp fiber and its composites—A review. J. Compos. Mater..

[26] Deitch R. (2003). Hemp: American History Revisited.

[27] Johnson R. Hemp as an Agricultural Commodity. https://fas.org/sgp/crs/misc/RL32725.pdf.

[28] Abuhasira R., Shbiro L., Landschaft Y. (2018). Medical use of cannabis and cannabinoids containing products-Regulations in Eu-rope and North America. Eur. J. Intern. Med..

[29] EFSA Panel on Additives and Products or Substances used in Animal Feed, (FEEDAP) (2011). Scientific opinion on the safety of hemp (Cannabis genus) for use as animal feed. EFSA J..

[30] Commission Regulation (EU) No 2017/1017 of 15 June 2017 Amending Regulation (EU) No 68/2013 on the Catalogue of Feed Materials. OJEU. 2017; Volume 159, pp. 48–119. https://eur-lex.europa.eu/legal-content/EN/TXT/?uri=CELEX%3A32017R1017.

[31] Vonapartis E., Aubin M., Seguin P., Mustafa A.F., Charron J.-B. (2015). Seed composition of ten industrial hemp cultivars approved for production in Canada. J. Food. Compost. Anal..

[32] Mierlita D. (2018). Effects of diets containing hempseeds or hemp cakes on fatty acid composition and oxidative stability of sheep milk. S. Afr. J. Anim. Sci..

[33] Devi V., Khanam S. (2019). Comparative study of different extraction processes for hemp (*Cannabis sativa*) seed oil considering physical, chemical and industrial-scale economic aspects. J. Clean. Prod..

[34] Yu L.L., Zhou K.K., Parry J. (2005). Antioxidant properties of cold-pressed black caraway, carrot, cranberry, and hemp seed oils. Food Chem..

[35] Atalay S., Jarocka-Karpowicz I., Skrzydlewska E. (2020). Antioxidative and Anti-Inflammatory Properties of Cannabidiol. Antioxi-dants.

[36] United Nations Office on Drugs and Crime. World Drug Report. 2005. https://www.unodc.org/unodc/en/data-and-analysis/WDR-2005.html.

[37] Moreno T., Montanes F., Tallon S.J., Fenton T., King J.W. (2020). Extraction of cannabinoids from hemp (*Cannabis sativa* L.) using high pressure solvents: An overview of different processing options. J. Supercrit. Fluids..

[38] Aladić K. (2015). Cold Pressing and Supercritical CO2 Extraction of Hemp (*Cannabis sativa*) Seed Oil. Chem. Biochem. Eng. Q..

[39] Whittle B.C.A., Hill I.R., Flockhart D.V.D., Gibson P., Whittle G.W.W., Brian A., Hill C.A., Flockhart I.R., Downs D.V., Gibson P. (2008). et al. Extraction of Pharmaceutically Active Components from Plant Materials. U.S. Patent.

[40] Rosenthal E. (2014). Beyond Buds: Marijuana Extracts-Hash, Vaping, Dabbing, Edibles & Medicines.

[41] Rutz A. (2016). CPC Distribution Chromatography of Cannabinoids. U.S. Patent.

[42] Soxhlet F. (1879). Scopus—Document details. Dinglers. Polytech. J..

[43] Jadhav D., Rekha B.N., Gogate P.R., Rathod V.K. (2009). Extraction of vanillin from vanilla pods: A comparison study of conven-tional soxhlet and ultrasound assisted extraction. J. Food Eng..

[44] Cheng H.F. (2012). Green Engineering: Green Composite Material, Biodiesel from Waster Coffee Grounds, and Polyurethane Bio-Foam.

[45] Nakahara Y., Sekine H. (1985). Studies on Confirmation of Cannabis Use. I. Determination of the Cannabinoid Contents in Mariju-ana Cigarette, Tar, and Ash Using High Performance Liquid Chromatography with Electrochemical Detection. J. Anal. Toxicol..

[46] Chawankul N., Chuaprasert S., Douglas P., Luewisutthichat W. (2001). Simulation of an agitated thin film evaporator for concen-trating orange juice using AspenPlus. J. Food Eng..

[47] Halim R., Danquah M.K., Webley P.A. (2012). Extraction of oil from microalgae for biodiesel production: A review. Biotechnol. Adv..

[48] Wang L., Weller C.L. (2006). Recent advances in extraction of nutraceuticals from plants. Trends Food Sci. Technol..

[49] Pandohee J., Holland B.J., Li B., Tsuzuki T., Stevenson P.G., Barnett N.W., Pearson J.R., Jones O.A.H., Conlan X.A. (2015). Screening of cannabinoids in industrial-grade hemp using two-dimensional liquid chromatography coupled with acidic po-tassium permanganate chemiluminescence detection. J. Sep. Sci..

[50] Chang C.W., Yen C.C., Wu M.T., Hsu M.C., Wu T.Y. (2017). Microwave-assisted extraction of cannabinoids in hemp nut using response surface methodology: Optimization and comparative study. Molecules.

[51] Herrero M., Cifuentes A., Ibañez E. (2006). Sub- and supercritical fluid extraction of functional ingredients from different natural sources: Plants, food-by-products, algae and microalgae—A review. Food Chem..

[52] Cunha V.M.B., da Silva M.P., da Costa W.A., de Oliveira M.S., Bezerra F.W.F., de Melo A.C., Pinto R.H.H., Machado N.T., Araujo M.E., de Junior R.N.C. (2018). Carbon Dioxide Use in High-Pressure Extraction Processes. Carbon Dioxide Chemistry, Cap-ture and Oil Recovery.

[53] Moslavac T., Joki´c S., Šubari´c D., Aladi´c K., Vukoja J., Prce N. (2014). Pressing and supercritical CO_2_ extraction of Camelina sativa oil. Ind. Crops. Prod..

[54] Rovetto L.J., Aieta N.V. (2017). Supercritical carbon dioxide extraction of cannabinoids from *Cannabis sativa* L.. J. Supercrit. Fluids..

[55] Smith R., Inomata H., Peters C. (2013). Introduction to Supercritical Fluids.

[56] Aladi´c K., Jarni K., Barbir T., Vidovi´c S., Vladi´c J., Bili´c M., Joki´c S. (2015). Supercritical CO_2_ extraction of hemp (*Cannabis sati-va* L.) seed oil. Ind. Crops. Prod..

[57] Plaza M., Turner C. (2015). Pressurized hot water extraction of bioactives. Trends. Analyt. Chem..

[58] Nuapia Y., Tutu H., Chimuka L., Cukrowska E. (2020). Selective extraction of cannabinoid compounds from cannabis seed using pressurized hot water extraction. Molecules.

[59] Nahar L., Onder A., Sarker S.D. (2020). A review on the recent advances in HPLC, UHPLC and UPLC analyses of naturally occur-ring cannabinoids (2010–2019). Phytochem. Anal..

[60] Gul W., Gul S.W., Radwan M.M., Wanas A.S., Mehmedic Z., Khan I.I., Sharaf M.H., ElSohly M.A. (2015). Determination of 11 cannabinoids in biomass and extracts of different varieties of cannabis using high-performance liquid chromatography. J. AOAC Int..

[61] Mandrioli M., Tura M., Scotti S., Toschi T.G. (2019). Fast detection of 10 cannabinoids by RP-HPLC-UV method in *Cannabis sativa* L.. Molecules.

[62] Madej K., Kózka G., Winiarski M., Piekoszewski W. (2020). A Simple, Fast, and Green Oil Sample Preparation Method for Deter-mination of Cannabidioloic Acid and Cannabidiol by HPLC-DAD. Separations.

[63] Salzet M., Stefano G.B. (2002). The endocannabinoid system in invertebrates. Prostaglandins Leukot. Essent. Fatty Acids.

[64] Oltrabella F., Melgoza A., Nguyen B., Guo S. (2017). Role of the endocannabinoid system in vertebrates: Emphasis on the zebrafish model. Dev. Growth Differ..

[65] Breivogel C.S., McPartland J.M., Parekh B. (2018). Investigation of non-CB 1, non-CB 2 WIN55212-2-sensitive G-protein-coupled receptors in the brains of mammals, birds, and amphibians. J. Recept. Signal Transduct. Res..

[66] Chiocchetti R., Galiazzo G., Tagliavia C., Stanzani A., Giancola F., Menchetti M., Militerno G., Bernardini C., Forni M., Mandrioli L. (2019). Cellular distribution of canonical and putative cannabinoid receptors in canine cervical dorsal root ganglia. Front. Vet. Sci..

[67] Dall’aglio C., Mercati F., Pascucci L., Boiti C., Pedini V., Ceccarelli P. (2010). Immunohistochemical localization of CB1 receptor in canine salivary glands. Vet. Res. Commun..

[68] Pirone A., Lenzi C., Coli A., Giannessi E., Stornelli M.R., Miragliotta V. (2015). Preferential epithelial expression of type-1 canna-binoid receptor (CB1R) in the developing canine embryo. SpringerPlus.

[69] Galiazzo G., Giancola F., Stanzani A., Fracassi F., Bernardini C., Forni M., Pietra M., Chiocchetti R. (2018). Localization of cannabinoid receptors CB1, CB2, GPR55, and PPARa in the canine gastrointestinal tract. Histochem. Cell Biol..

[70] Gebremedhin D., Lange A.R., Campbell W.B., Hillard C.J., Harder D.R. (1999). Cannabinoid CB1 receptor of cat cerebral arterial muscle functions to inhibit L-type Ca^2+^ channel current. Am. J. Physiol..

[71] Miragliotta V., Ricci P.L., Albanese F., Pirone A., Tognotti D., Abramo F. (2018). Cannabinoid receptor types 1 and 2 and peroxi-some proliferatoractivated receptor-a: Distribution in the skin of clinically healthy cats and cats with hypersensitivity derma-titis. Vet. Dermatol..

[72] Pirone A., Lenzi C., Briganti A., Abbate F., Levanti M., Abramo F., Miragliotta V. (2017). Spatial distribution of cannabinoid receptor 1 and fatty acid amide hydrolase in the cat ovary and oviduct. Acta Histochem..

[73] Corroon J., Felice J.F. (2019). The endocannabinoid system and its modulation by cannabidiol (CBD). Altern. Ther. Health Med..

[74] Kumar A., Premoli M., Aria F., Bonini S.A., Maccarinelli G., Gianoncelli A., Memo M., Mastinu A. (2019). Cannabimimetic plants: Are they new cannabinoidergic modulators?. Planta.

[75] Andre C.M., Hausman J.F., Guerriero G. (2016). Cannabis sativa: The plant of the thousand and one molecules. Front. Plant Sci..

[76] Mastinu A., Premoli M., Ferrari-Toninelli G., Tambaro S., Maccarinelli G., Memo M., Bonini S.A. (2018). Cannabinoids in health and disease: Pharmacological potential in metabolic syndrome and neuroinflammation. Horm. Mol. Biol. Clin. Investig..

[77] Han K.H., Lim S., Ryu J., Lee C.W., Kim Y., Kang J.H., Kang S.S., Ahn Y.K., Park C.S., Kim J.J. (2009). CB1 and CB2 cannabinoid receptors differentially regulate the production of reactive oxygen species by macrophages. Cardiovasc. Res..

[78] Massi P., Vaccani A., Ceruti S., Colombo A., Abbracchio M.P., Parolaro D. (2004). Antitumor effects of cannabidiol, a nonpsycho-active cannabinoid, on human glioma cell lines. J. Pharmacol. Exp. Ther..

[79] Sánchez C., de Ceballos M.L., Del Pulgar T.G., Rueda D., Corbacho C., Velasco G., Galve-Roperh I., Human J.W., Cajal S.R.Y., Guzmán M. (2001). Inhibition of glioma growth in vivo by selective activation of the CB (2) cannabinoid receptor. Cancer Res..

[80] Freeman A.M., Petrilli K., Lees R., Hindocha C., Mokrysz C., Curran H.V., Saunders R., Freeman T.P. (2019). How does canna-bidiol (CBD) influence the acute effects of delta-9-tetrahydrocannabinol (THC) in humans? A systematic review. Neurosci. Bi-obehav. Rev..

[81] Mlost J., Bryk M., Starowicz K. (2020). Cannabidiol for Pain Treatment: Focus on Pharmacology and Mechanism of Action. Int. J. Mol. Sci..

[82] Tham M., Yilmaz O., Alaverdashvili M., Kelly M.E.M., Denovan-Wright E.M., Laprairie R.B. (2019). Allosteric and orthosteric pharmacology of cannabidiol and cannabidiol- dimethylheptyl at the type 1 and type 2 cannabinoid receptors. Br. J. Pharma-col..

[83] Gallelli C.A., Calcagnini S., Romano A., Koczwara J.B., De Ceglia M., Dante D., Villani R., Giudetti A.M., Cassano T., Gaetani S. (2018). Modulation of the Oxidative Stress and Lipid Peroxidation by Endocannabinoids and Their Lipid Analogues. An-tioxidants.

[84] O’Sullivan S.E. (2016). An update on PPAR activation by cannabinoids. Br. J. Pharmacol..

[85] Bujak J.K., Kosmala D., Szopa I.M., Majchrzak K., Bednarczyk P. (2019). Inflammation, Cancer and Immunity—Implication of TRPV1 Channel. Front. Oncol..

[86] Hartsel J.A., Boyar K., Pham A., Silver R.J., Makriyannis A., Gupta R., Srivastava A., Lall R. (2019). Cannabis in veterinary medicine: Cannabinoid therapies for animals. Nutraceuticals in Veterinary Medicine.

[87] Kogan L., Schoenfeld-Tacher R., Hellyer P., Rishniw M. (2019). US veterinarians’ knowledge, experience, and perception regarding the use of Cannabidiol for canine medical conditions. Front. Vet. Sci..

[88] Taylor L., Gidal B., Blakey G., Tayo B., Morrison G. (2018). A Phase I, Randomized, Double-Blind, Placebo-Controlled, Single As-cending Dose, Multiple Dose, and Food Effect Trial of the Safety, Tolerability and Pharmacokinetics of Highly Purified Can-nabidiol in Healthy Subjects. CNS Drugs.

[89] Devinsky O., Patel A.D., Thiele E.A., Wong M.H., Appleton R., Harden C.L., Greenwood S., Morrison G., Sommerville K. (2018). Randomized, dose-ranging safety trial of cannabidiol in Dravet syndrome. Neurology.

[90] Manini A.F., Yiannoulos G., Bergamaschi M.M., Hernandez S., Olmedo R., Barnes A.J., Winkel G., Sinha R., Jutras-Aswad D., Huestis M.A. (2015). Safety and pharmacokinetics of oral Cannabidiol when administered concomitantly with intravenous Fentanyl in humans. J. Addict. Med..

[91] Gamble L.J., Boesch J.M., Frye C.W., Schwark W.S., Mann S., Wolfe L., Brown H., Berthelsen E.S., Wakshlag J.J. (2018). Pharmacokinetics, safety, and clinical efficacy of cannabidiol treatment in osteoarthritic dogs. Front. Vet. Sci..

[92] Kogan L., Hellyer P., Downing R. The Use of Cannabidiol-Rich Hemp Oil Extract to Treat Canine Osteoarthritis-Related Pain: A Pilot Study. https://www.researchgate.net/publication/339698157_The_Use_of_Cannabidiol-Rich_Hemp_Oil_Extract_to_Treat_Canine_Osteoarthritis_Related_Pain_A_Pilot_Study.

[93] Verrico C.D., Wesson S., Konduri V., Hofferek C.J., Vazquez-Perez J., Blair E., Dunner K., Salimpour P., Decker W.K., Halpert M.M. (2020). A Randomized, Double-Blind, Placebo-Controlled Study of Daily Cannabidiol for the Treatment of Canine Osteoarthritis Pain. Pain.

[94] Hammell D.C., Zhang L.P., Ma F., Abshire S.M., McIlwrath S.L., Stinchcomb A.L., Westlund K.N. (2016). Transdermal cannabidi-ol reduces inflammation and pain-related behaviours in a rat model of arthritis. Eur. J. Pain.

[95] De Gregorio D., McLaughlin R.J., Posa L., Ochoa-Sanchez R., Enns J., Lopez-Canul M., Aboud M., Maione S., Comai S., Gobbi G. (2019). Cannabidiol modulates serotonergic transmission and reverses both allodynia and anxiety-like behavior in a mod-el of neuropathic pain. Pain.

[96] Maione S., Piscitelli F., Gatta L., Vita D., De Petrocellis L., Palazzo E., De Novellis V., Di Marzo V. (2011). Non-psychoactive can-nabinoids modulate the descending pathway of antinociception in anaesthetized rats through several mechanisms of action. Br. J. Pharmacol..

[97] Abraham A.D., Leung E.J.Y., Wong B.A., Rivera Z.M.G., Kruse L.C., Clark J.J., Land B.B. (2020). Orally consumed cannabinoids provide long-lasting relief of allodynia in a mouse model of chronic neuropathic pain. Neuropsychopharmacology.

[98] Lehmann C., Fisher N.B., Tugwell B., Szczesniak A., Kelly M., Zhou J. (2016). Experimental cannabidiol treatment reduces early pancreatic inflammation in type 1 diabetes. Clin. Hemorheol. Microcirc..

[99] Li H., Kong W., Chambers C.R., Yu D., Ganea D., Tuma R.F., Ward S.J. (2018). The non-psychoactive phytocannabinoid canna-bidiol (CBD) attenuates pro-inflammatory mediators, T cell infiltration, and thermal sensitivity following spinal cord injury in mice. Cell. Immunol..

[100] Xu D.H., Cullen B.D., Tang M., Fang Y. (2019). The Effectiveness of Topical Cannabidiol Oil in Symptomatic Relief of Peripheral Neuropathy of the Lower Extremities. Curr. Pharm. Biotechnol..

[101] Szaflarski J.P., Bebin E.M., Cutter G., DeWolfe J., Dure L.S., Gaston T.E., Kankirawatana P., Liu Y., Singh R., Standaert D.G. (2018). Cannabidiol improves frequency and severity of seizures and reduces adverse events in an open-label add-on prospective study. Epilepsy Behav..

[102] Laux L.C., Bebin E.M., Checketts D., Chez M., Flamini R., Marsh E.D., Miller I., Nichol K., Park Y., Segal E. (2019). Long-term safety and efficacy of cannabidiol in children and adults with treatmentresistant Lennox-Gastaut syndrome or Dravet syndrome: Expanded access program results. Epilepsy Res..

[103] Devinsky O., Cross J.H., Laux L., Marsh E., Miller I., Nabbout R., Scheffer I.E., Thiele E.A., Wright S. (2017). Trial of cannabidiol for drug-resistant seizures in the dravet syndrome. N. Engl. J. Med..

[104] Choy E. (2012). Understanding the dynamics: Pathways involved in the pathogenesis of rheumatoid arthritis. Rheumatology.

[105] Julian R.J. (1998). Rapid Growth Problems: Ascites and Skeletal Deformities in Broilers. Poult. Sci..

[106] Sandilands V. (2011). The laying hen and bone fractures. Vet. Rec..

[107] Gakhar N., Goldberg E., Jing M., Gibson R., House J.D. (2012). Effect of feeding hemp seed and hemp seed oil on laying hen per-formance and egg yolk fatty acid content: Evidence of their safety and efficacy for laying hen diets. Poult. Sci..

[108] Neijat M., Gakhar N., Neufeld J., House J.D. (2014). Performance, egg quality, and blood plasma chemistry of laying hens fed hempseed and hempseed oil. Poult. Sci..

[109] Skřivan M., Englmaierova M., Taubner T., Skřivanova E. (2020). Effects of Dietary Hemp Seed and Flaxseed on Growth Perfor-mance, Meat Fatty Acid Compositions, Liver Tocopherol Concentration and Bone Strength of Cockerels. Animals.

[110] Vispute M.M., Sharma D., Mandal A.B., Rokade J.J., Tyagi P.K., Yadav A.S. (2019). Effect of dietary supplementation of hemp (Cannabis sativa) and dill seed (*Anethum graveolens*) on performance, serum biochemicals and gut health of broiler chickens. J. Anim. Physiol. Anim. Nutr..

[111] Lekanich C.O., Noble R.C. (1997). Manipulation of the n-3 polyunsaturated fatty acid composition of avian eggs and meat. Worlds. Poult. Sci. J..

[112] Hermier D. (1997). Lipoprotein Metabolism and Fattening in Poultry. J. Nutr..

[113] Bensadoun A., Rothfeld A. (1972). The Form of Absorption of Lipids in the Chicken, Gallus domesticus. Proc. Soc. Exp. Biol. Med..

[114] Jiang S., Cui L., Shi C., Ke X., Luo J., Hou J. (2013). Effects of dietary energy and calcium levels on performance, egg shell quality and bone metabolism in hens. Vet. J..

[115] Fernandez A., Verde M.T., Gascon M., Ramos J., Gomez J., Luco D.F., Chavez D. (1994). Variations of clinical biochemical param-eters of laying hens and broiler chickens fed aflatoxin-containing feed. Avian Pathol..

[116] Diaz G.J., Squires E.J., Julian R.J. (1999). The Use of Selected Plasma Enzyme Activities for the Diagnosis of Fatty LiverHemorrhag-ic Syndrome in Laying Hens. Avian Dis..

[117] McGrath S., Bartner L.R., Rao S., Pacher R.A., Gustafson D.L. (2019). Randomized blinded controlled clinical trial to assess the ef-fect of oral cannabidiol administration in addition to conventional antiepileptic treatment on seizure frequency in dogs with intractable idiopathic epilepsy. J. Am. Vet. Med. Assoc..

[118] Deabold K.A., Schwark W.S., Wolf L., Wakshlag J.J. (2019). Single-Dose Pharmacokinetics and preliminary safety assessment with use of CBD rich Hemp nutraceutical in healthy dogs and cats. Animals.

[119] Ewing L.E., Skinner C.M., Quick C.M., Kennon-McGill S., McGill M.R., Walker L.A., ElSohly M.A., Gurley B.J., Kotur-bash I. (2019). Hepatotoxicity of a Cannabidiol-Rich Cannabis Extract in the Mouse Model. Molecules.

[120] Samara E., Brown N.K., Harvey D.J. (1990). Microsomal metabolism of the 1”,1”-dimethylheptyl analogue of cannabidiol: Relative percentage of monohydroxy metabolites in four species. Drug Metab. Dispos..

[121] Bornheim L.M., Everhart E.T., Li J., Almira Correia M. (1994). Induction and genetic regulation of mouse hepatic cytochrome P450 by cannabidiol. Biochem. Pharmacol..

[122] Grotenhermen F., Muller-Vahl K. (2016). Cannabis und Cannabinoide in der Medizin: Fakten und Ausblick. Suchttherapie.

[123] Castillo A., Tolón M.R., Fernández-Ruiz J., Romero J., Martinez-Orgado J. (2010). The neuroprotective effect of cannabidiol in an in vitro model of newborn hypoxic-ischemic brain damage in mice is mediated by CB(2) and adenosine receptors. Neurobiol. Dis..

[124] Mori M.A., Meyer E., Soares L.M., Milani H., Guimarães F.S., de Oliveira R.M.W. (2017). Cannabidiol reduces neuroinflammation and promotes neuroplasticity and functional recovery after brain ischemia. Prog. Neuropsychopharmacol. Biol. Psychiatry.

[125] Bitencourt R.M., Takahashi R.N. (2018). Cannabidiol as a therapeutic alternative for post-traumatic stress disorder: From bench research to confirmation in human trials. Front. Neurosci..

[126] Shbiro L., Hen-Shoval D., Hazut N., Rapps K., Dar S., Zalsman G., Mechoulam R., Weller A., Shoval G. (2019). Effects of canna-bidiol in males and females in two different rat models of depression. Physiol. Behav..

[127] Salim S. (2017). Oxidative Stress and the Central Nervous System. J. Pharmacol. Exp. Ther..

[128] Peres F.F., Lima A.C., Hallak J.E.C., Crippa J.A., Silva R.H., Abílio V.C. (2018). Cannabidiol as a Promising Strategy to Treat and Prevent Movement Disorders?. Front. Pharmacol..

[129] Costa B., Trovato A.E., Comelli F., Giagnoni G., Colleoni M. (2007). The non-psychoactive cannabis constituent cannabidiol is an orally effective therapeutic agent in rat chronic inflammatory and neuropathic pain. Eur. J. Pharmacol..

[130] Juknat A., Pietr M., Kozela E., Rimmerman N., Levy R., Gao F., Coppola G., Geschwind D., Vogel Z. (2013). Microarray and pathway analysis reveal distinct mechanisms underlying cannabinoid-mediated modulation of LPS-induced activation of BV-2 microglial cells. PLoS ONE.

[131] Vomund S., Schäfer A., Parnham M.J., Brüne B., von Knethen A. (2017). Nrf2, the Master Regulator of Anti-Oxidative Responses. Int. J. Mol. Sci..

[132] Borges R.S., Batista J., Viana R.B., Baetas A.C., Orestes E., Andrade M.A., Honório K.M., Da Silva A.B. (2013). Understanding the Molecular Aspects of Tetrahydrocannabinol and Cannabidiol as Antioxidants. Molecules.

[133] Wu H.Y., Goble K., Mecha M., Wang C.C., Huang C.H., Guaza C., Jan T.R. (2012). Cannabidiol-induced apoptosis in murine mi-croglial cells through lipid raft. GLIA.

[134] Gęgotek A., Ambrożewicz E., Jastrząb A., Jarocka-Karpowicz I., Skrzydlewska E. (2019). Rutin and ascorbic acid cooperation in antioxidant and antiapoptotic effect on human skin keratinocytes and fibroblasts exposed to UVA and UVB radiation. Arch. Dermatol. Res..

[135] Gaschler M.M., Stockwell B.R. (2017). Lipid peroxidation in cell death. Biochem. Biophys. Res. Commun..

[136] Ayala A., Muñoz M.F., Argüelles S. (2014). Lipid peroxidation: Production, metabolism, and signaling mechanisms of malondial-dehyde and 4-hydroxy-2-nonenal. Oxid. Med. Cell. Longev..

[137] Sun S., Hu F., Wu J., Zhanga S. (2017). Cannabidiol attenuates OGD/R-induced damage by enhancing mitochondrial bioenergetics and modulating glucose metabolism via pentose-phosphate pathway in hippocampal neurons. Redox Biol..

[138] Bih C.I., Chen T., Nunn A.V.W., Bazelot M., Dallas M., Whalley B.J. (2015). Molecular Targets of Cannabidiol in Neurological Dis-orders. Neurotherapeutics.

[139] Wang Y., Mukhopadhyay P., Cao Z., Wang H., Feng D., Haskó G., Mechoulam R., Gao B., Pacher P. (2017). Cannabidiol attenu-ates alcohol-induced liver steatosis, metabolic dysregulation, inflammation and neutrophil-mediated injury. Sci. Rep..

[140] Jing M., Zhao S., House J.D. (2017). Performance and tissue fatty acid profile of broiler chickens and laying hens fed hemp oil and HempOmegaTM. Poult. Sci..

[141] Kasula R., Solis F., Shaffer B., Connett F., Barrett C., Cocker R., Willinghan E. (2021). Effect of dietary hemp seed cake on the per-formance of commercial laying hens. Int. J. Livest. Prod..

[142] Kasula R., Solis F., Shaffer B., Connett F., Barrett C., Cocker R., Willinghan E. (2021). Hemp seed cake increases fatty acids but does not transfer cannabinoids in eggs and tissues of laying hens. Int. J. Livest. Prod..

[143] Skřivan M., Englmaierova´ M., Vı´t T., Skřivanova E. (2019). Hempseed increases gamma-tocopherol in egg yolks and the breaking strength of tibias in laying hens. PLoS ONE.

[144] Konieczka P., Szkopek D., Kinsner M., Fotschki B., Juśkiewicz J., Banach J. (2020). Cannabis-derived cannabidiol and nanoseleni-um improve gut barrier function and affect bacterial enzyme activity in chickens subjected to C. perfringens challenge. Vet. Res..

[145] Mahmoudi M., Farhoomand P., Nourmohammadi R. (2015). Effects of different levels of hempseed (*Cannabis sativa* L.) and dextran oligosaccharide on growth performance and antibody titer response of broiler chickens. Ital. J. Anim. Sci..

[146] Yalcin H., Konca Y., Durmuscelebi F. (2018). Effect of dietary supplementation of hemp seed (Cannabis sativa L.) on meat quality and egg fatty acid composition of Japanese quail (*Coturnix coturnix* japonica). J. Anim. Physiol. Anim. Nutr..

[147] Karlsson L., Finell M., Martinsson K. (2010). Effects of increasing amounts of hempseed cakes in the diet of dairy cows on the pro-duction and composition of milk. Animal.

[148] Gibb D.J., Shah M.A., Mir P.S., McAllister T.A. (2005). Effect of full-fat hemp seed on performance and tissue fatty acids of feedlot cattle. Can. J. Anim. Sci..

[149] Kleinhenz M.D., Weeder M., Montgomery S., Martin M., Curtis A., Magnin G., Lin Z., Griffin J., Coetzee J.F. (2022). Short term feeding of industrial hemp with a high cannabidiolic acid (CBDA) content increases lying behavior and reduces biomarkers of stress and inflammation in Holstein steers. Sci. Rep..

[150] Kleinhenz M.D., Magnin G., Lin Z., Griffin J., Kleinhenz K.E., Montgomery S., Curtis A., Martin M., Coetzee J.F. (2020). Plasma concentrations of eleven cannabinoids in cattle following oral administration of industrial hemp (*Cannabis sativa*). Sci. Rep..

[151] Cozma A., Andrei S., Pintea A., Miere D., Filip L., Loghin F., Ferlay A. (2015). Effect of hemp seed oil supplementation on plasma lipid profile, liver function, milk fatty acid, cholesterol, and vitamin A concentrations in Carpathian goats. Czech J. Anim. Sci..

[152] Palade L.M., Habeanu M., Marin D.M., Sanda Chedea V., Cecilia Pistol G., Alexandru Grosu I., Gheorghe A., Ropota M., Taranu I. (2019). Effect of Dietary Hemp Seed on Oxidative Status in Sows during Late Gestation and Lactation and Their Offspring. Animals.

